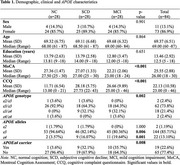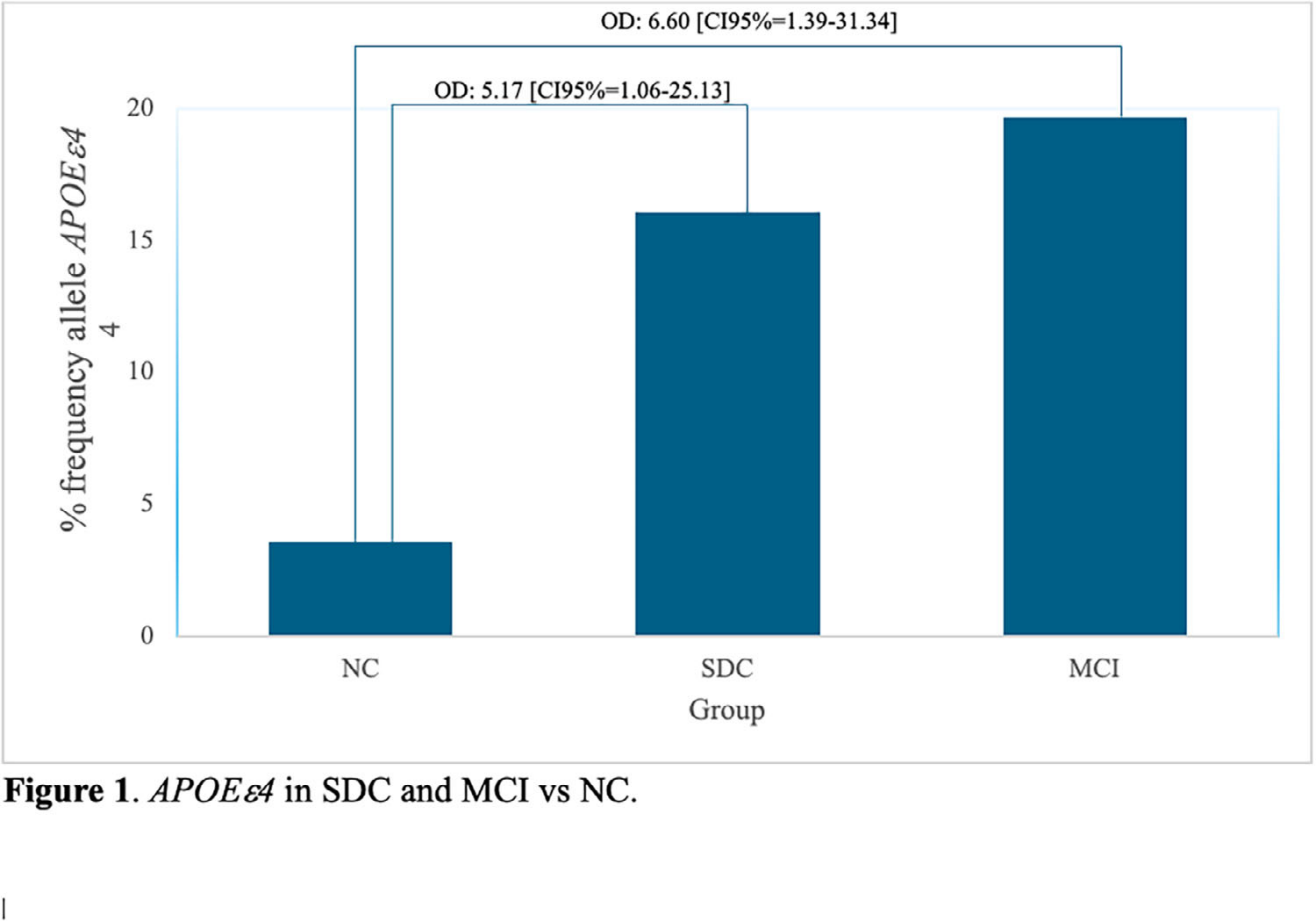# Association of the APOEε*4* allele in Mexican‐mestizo older adults with subjective cognitive decline and mild cognitive impairment

**DOI:** 10.1002/alz70857_101299

**Published:** 2025-12-25

**Authors:** Ángela Acosta‐Amaya, Salvador Sánchez‐Badajos, Diego Solano‐Mendoza, Nancy Monroy‐Jaramillo, Alberto Ortega‐Vázquez, Fernanda Infante‐Esquivel, David J. Dávila‐Ortiz de Montellano, Ramiro Ruiz‐García, Yaneth Rodríguez‐Agudelo

**Affiliations:** ^1^ Universidad Nacional Autónoma de México, México, EM, Mexico; ^2^ Instituto Nacional de Neurología y Neurocirugía Manuel Velasco Suárez, México, EM, Mexico; ^3^ Universidad Autónoma Metropolitana, Unidad Xochimilco, México, EM, Mexico; ^4^ Universidad Autónoma Metropolitana (UAM), Unidad Xochimilco, México, EM, Mexico; ^5^ Instituto Nacional de Neurología y Neurocirugía Manuel Velasco Suárez (INNNMVS), México, EM, Mexico; ^6^ Instituto Nacional de Neurología y Neurocirugía Manuel Velasco Suárez (INNNMVS), Mexico, EM, Mexico; ^7^ Instituto Nacional de Neurología y Neurocirugía Manuel Velasco Suárez (INNNMVS), Mexico City, EM, Mexico

## Abstract

**Background:**

Subjective cognitive decline (SCD) is a state in which individuals complain of cognitive impairment; however, they perform normally on standard neuropsychological tests. SCD is considered a diagnostic entity of risk for mild cognitive impairment (MCI) and dementia. SCD and MCI correspond to stages 2 and 3, respectively, in the clinical staging of the continuum of the Alzheimer's disease (AD). Portability of *APOEε4* allele increases AD's risk, and its interaction with SCD might raise the cognitive impairment's risk. Herein, we aimed to explore the relationship between SCD, MCI and *APOEε4* status in Mexican‐mestizo older adults.

**Method:**

84 Mexican‐mestizo older adults (86.9% females) were included after consent was obtained (protocol INNN_139/23). Three comparable groups by age (69.37±6.51 y.o.) and years of schooling (13.46±2.92) were formed, *n* = 28/group, as follows: participants with normal cognition (NC), SCD and MCI. The MoCA and the Cognitive Complaint Questionnaire (CCQ) cut point scores at enrollment were used to classify the groups. *APOE* genotyping was assessed by allelic discrimination and SPSS was used for statistical analysis.

**Result:**

Significant differences were observed in the cognitive performance between the NC and MCI groups (27.36±1.47 vs. 22.21± 2.06, *p* <0.001). Regarding the cognitive complaint, NC group showed the lowest score, followed by the DCS and MCI groups (11.71± 6.54, 28.18± 5.75 and 26.66± 9.89, *p* <0.001) (Table 1). The distribution of *APOE* genotype and allele frequencies of the studied sample was within Hardy–Weinberg equilibrium. Comparison of *APOEε4* allele frequency was different between NC vs. SCD and MCI (*p* <0.005), while SCD and MCI groups behaved similarly (*p* >0.05). Being carrier of *APOEε4* increased the risk of SCD (OR=5.17, CI95%=1.06‐25.13, *p* = 0.04) and MCI (OR=6.60, CI95%=1.39‐31.34, *p* = 0.02), compared to the NC group (Figure 1).

**Conclusion:**

An association between SCD, MCI and *APOEε4* was identified in our sample of mestizo‐Mexican older adults; of note, no differences were found for *APOEε4* allele between the SCD and MCI groups. The risk of portability of *APOEe4* was comparable in SCD and MCI, highlighting SCD as a clinical risk prediction entity in the AD continuum. The relevance of SCD should be further studied in the prevention of dementia by improving early detection.